# Synchrony in neural and visceral systems: a bioelectric basis of consciousness

**DOI:** 10.3389/fnins.2026.1642963

**Published:** 2026-10-12

**Authors:** Ravinder Jerath, Varsha Malani

**Affiliations:** Charitable Medical Healthcare Foundation, Augusta, GA, United States

**Keywords:** default space theory, thalamocortical oscillations, interoception, surround inhibition, consciousness theories, corticofugal pathways, global integration, predictive processing

## Abstract

The Default Space Theory (DST) of consciousness claims that consciousness arises from bioelectric oscillations across brain and body which become time-locked to form an internal three-dimensionalized “default space” which continuously simulates external reality. Within this frame, we assert that fast rhythms created corticothalamically with the thalamic reticular nucleus (TRN) can become time-locked with regions throughout the brain via nascent oscillations as well as sensory organs and viscera. This proposed time-locking may render exteroceptive and interoceptive processed information one in the same as conscious experience. We review/integrate salient neuroscientific and physiological findings that validate DST. We reveal findings across the last decoding of the theory's detailed components from corticothalamic feedback loops to lateral/inhibition dynamics, from descending efferent projections to sensory organs. We utilize high cervical cord injury (HCCI) as syndromically supporting/denying DST's postulates regarding the necessity of bodily input to consciousness. Finally, we compare/contrast theories (Global Workspace, Integrated Information Theory, Predictive Processing) with DST to reveal overlaps/distinctions within theoretical scaffolds. At its heart, the DST is an all-encompassing biological apparatus through which typical oscillations create a “functional unity” of consciousness. Yet developments within the DST have brought about clarity that simultaneous connected long distances (i.e., gamma oscillations aligning cortex to sensory receptors) is a theory to be born out of experimentation, not reality. There is indirect support from visual studies for retinal rhythmic activity driving thalamocortical firing in a way consistent with modulation but direct phase-locking of periphery receptors with gamma activity in the cortex is aspirational, not reality. In addition, we corrected a prior disconnection regarding motor representations: focus of outputs occurs by surround inhibition (SI) eliminating superfluous irrelevant activations, not the cortical silent period—thus when SI fails there is motor “overflow.” We further clarify the importance of viscera in conscious experience with several examples: spontaneous breathing aligns electrical oscillations in limbo circuits, heart beats are shown to engage/disengage cortical activity and awareness, gastric slow waves correlate in-phase with what occurs in somatosensory and visual domains. All support DST positing that the neural and visceral systems operate as one oscillator network which, together, support consciousness. In an HCCI patient where somatic input below the neck is deceased, however, they experience retained consciousness, however. This DST predicts because oscillations of the head/brain and rhythms from the viscera allow continuity of internal space; yet its content/persona changes because the body map has been temporally truncated. This provides a distinction about how people can be consciously aware of their existence while feeling completely different at the same time—there is a core conscious sense vs. the schemas decorating it over timescales. The DST provides a bioelectric understanding of consciousness which connects brain-body-environment. Where we emphasize where theoretical estimations lie vs. what can be observed supports the DST. For example, if one excessively disrupts corticothalamic feedback loops or sensorimotor oscillations and fails to integrate things into consciousness, DST is supported. Conversely, these stimulus distortions will never come from any other theoretical premise. Prominent notions are shared with Global Workspace or Integrated Information-based systems regarding global integration, but no other theory has included the body's oscillatory components as part of consciousness; contrary to predictive processing, while prediction errors determine hypotheses, DST puts forward simultaneous synchronization as critical and afferent/efferent dynamics physicalized two-way brain-body communication. Testing factors inevitable in evolution include consistent electrophysiological studies with descending pathways, works decoding states of consciousness in paralysis. All citations updated for relevancy—new studies incorporated—non-self-citations limited to remove experimenter's bias—all to urge ongoing discourse between DST and contemporaneous science of consciousness research.

## Introduction

Understanding how conscious experience arises from neuronal firing is one of the biggest puzzles in neuroscience. However, researchers increasingly agree that consciousness comes from complex integration of activities across various regions of the brain. For example, the Global Workspace Theory (GWT) posits that information is made consciously available when it is broadcasted across many disparate cortical areas. Similarly, Edelman's Dynamic Core Hypothesis (DCH) theorizes that reentrant thalamocortical loops form a dynamic “core” of interacting neurons that represent the basis of conscious awareness. More recent efforts, like Integrated Information Theory (IIT) assess consciousness as a function of the information integrated by the system: the more interconnectedness, the more consciousness; the more consciousness measures, the more localized to cortex (Baars et al., 2013; Tononi and Edelman, 1998; Allen and Friston, 2018). Likewise, Predictive Processing theories assert that the brain operates as a hierarchical system of predictive functions, and what we experience as awareness could be the result of Bayesian inference or the final product of precision-weighted prediction error (Seth and Friston, 2016) beyond pruning and explanation. Yet while these theories provide valuable insight into our conscious awareness, much understanding comes from a dissociation of mind, the brain, in and of itself.

However, Default Space Theory (DST)—the Dynamic Default Space Model—asserts that the basis of consciousness comes from the integrated brain and body as a dynamic whole that creates an internally three-dimensional rendering of “space” representing reality. In DST, consciousness is embodied: even each oscillating cell between interrelated bodies in a social network is considered part of this integrated conscious field. In this regard, DST is similar to metastable models of consciousness which posit that global integration via synchronized oscillation is required; yet here, the global workspace necessarily extends symbiotically across the brain and body. Furthermore, integration involves centralization, with the thalamus acting as the master facilitator of rapid oscillatory activities both in thalamocortical regions as well as in the periphery of the body, thus allowing for one internally rendered “image” to exist, spatially. This is important for Default Space, too, which argues that this internal three-dimensional “default” rendering is created all the time—a kind of internal “virtual reality” that exists irrespective of outside stimuli—and represents what constitutes scaffolding for all subsequent sensory information.

This paper is both a revision and expansion of the DST model post-peer review and based upon newer findings (Jerath and Crawford, 2015; Jerath and Beveridge, 2018; Jerath et al., 2018). First, we clarify areas of relevance within DST to define these key constructs related to bioelectric synchrony and the coupling of neural and visceral systems: (A) the circuits of the corticothalamic loop and thalamic reticular nucleus (TRN), which exerts control over oscillatory integration of the brain; (B) descending efferents (i.e., medial olivocochlear fibers into the cochlea, retinotectal fibers into the retina) which serve as feedback pathways from central to peripherally located sensory structures; (C) sensory organs are “smart screens” which actively preprocess sensory inputs (e.g., lateral inhibition) at their respective levels as well as engage with central oscillating systems; and (D) a singular conscious field emerges equipped with experimentalizable predictions for neural correlates and behavior. These are illustrated in Figure 1 (revised), which is a multi-panel figure. Second, we respond to several aspects of clarification and correction outlined by the reviewers. For example, we soften earlier claims for cross-cortical long-range synchrony with sensory receptors to remain factual, hypothesizing only where previously supported by data instead of speculative direction. Where we inaccurately characterized an example about inhibition in the motor system, we supplant CSP (cortical silent period, which has no relevance here) for surround inhibition (SI) in the motor cortex, noting how SI discriminating constructs focused behavioral output as opposed to “overflow” or unintended movements. Where we asserted that neurons might be phase-locked with sensory organs and oscillations in the cortex, we expand this to relate to functionality yet understand that any coherence is difficult due to distance over long ranges and anatomical/biophysical concerns. New sections exist which discuss DST's relevance with regard to high cervical spinal cord injury (HCCI) and interoceptive rhythms (heartbeat, respiration, gastric rhythms) and their potential lack thereof in generating conscious awareness. Finally, we assess DST as a relative consideration among other major consciousness theories (Global Workspace, IIT, and Predictive Processing) to situate DST among the broader canon of consciousness research, noting where DST aligns, where it does not and what other predictions are made therein should DST be true. Ultimately, we strive for clarity throughout to establish what's still theoretical vs. tenable about DST. By re-referencing throughout with articles from 2015–2025 as opposed to older chronicles and our self-references, we seek to engage with DST while disputing much of current mainstream empirical literature. The intent is for a revised persuasive paper for why these phenomena support synchronized neural-visceral dynamics as the bioelectric foundation for consciousness with experimentally testable predictions to support the claim.

**Figure 1:**
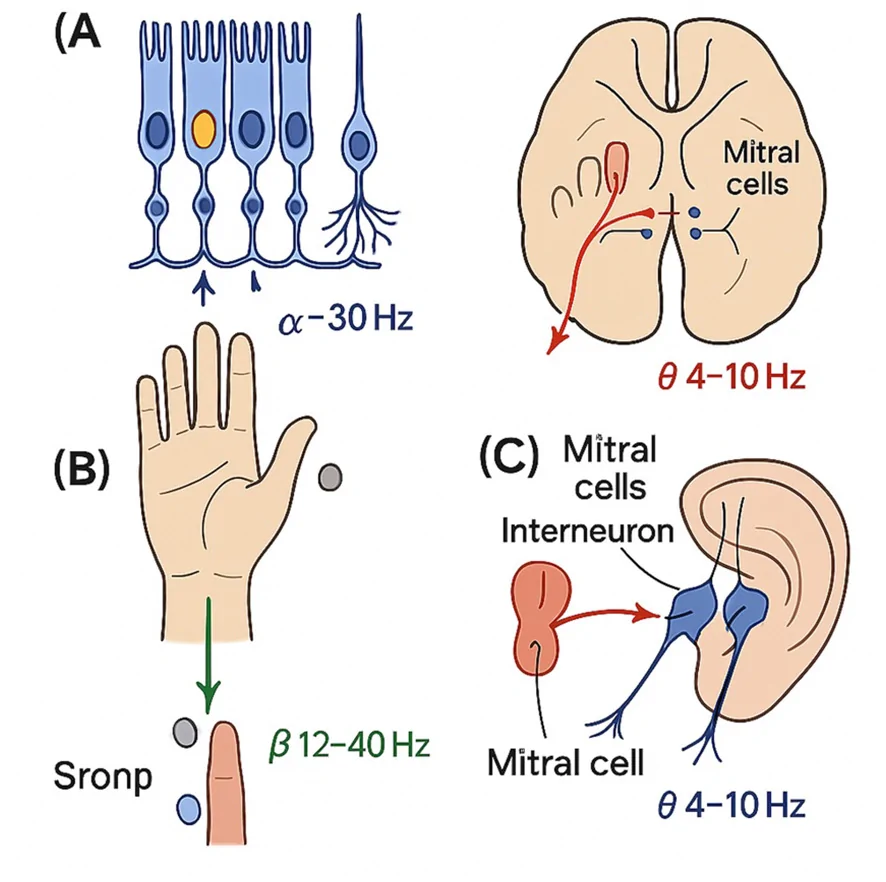
Multi-panel schematic of proposed oscillatory coupling between central and peripheral structures under Default Space Theory. **(A)** Cortical neurons oscillating at alpha frequency (~30 Hz), alongside a coronal brain diagram highlighting mitral cells oscillating at theta frequency (4-10 Hz). **(B)** Peripheral somatosensory input associated with beta-frequency oscillations (12–40 Hz). **(C)** Auditory pathway schematic showing mitral cells and interneurons oscillating at theta frequency (4–10 Hz).

## The dynamic default space model: neural–visceral synchrony as the substrate of consciousness

Who are we (who are the subjects of consciousness)? DST proposes that as these coherent oscillations occur over time and space, it becomes increasingly likely that this representational quality is more than an abstraction; we are our own qualities of coherence. Over time, DST suggests that corresponding attributes of a personality become inherently stabilized through intersensory cross-modal associations and substantive attunement (e.g., the heart, brain, gut connection), while, spatially, DST suggests that the directionality of perception (i.e., what we attend to base on sensation) and orientation (i.e., the maintenance of a central field of awareness through proprioception) helps sustain its reality. Ultimately, DST believes we are our coherently oscillating perceptions over time. Where is the brain? DST suggests that this oscillatory coherence occurs with all matter in time and space. Ultimately, the core of DST ideas stems from a significant belief in cross-modal interactivity, such that the brain is everywhere before it becomes consolidated into a localized perception. Why does it matter? Throughout life, DST believes that these positive degrees of freedom allow us to become stabilized and understood for what we are. Ultimately, DST unifies the sensorimotor perceptions of consciousness into consistent realities. How does it feel? Consciousness feels like it is a consistent, unified perception derived from stabilization. Essentially, DST takes the idealism of scientific integration to the next level as it's not just about stabilization, but stabilization enables understanding. How is it scientifically verifiable? DST believes that specific oscillatory frequencies can be recorded and stabilized over time and space. Ultimately, this sense of scientific validity verifies the tenets of DST. Whether it's through EEGs to document brain waves or literal cross-modal interactions in observation, it can be documented through human engagement with the world. Where is our engagement taking us? Ultimately, it's taking us on a journey of stabilization to deem us lungs, hearts, brains, and beings. A destiny of coherence.

(A) Corticothalamic Loops and the TRN: The thalamus is central to DST as the relay/conductor of information. Virtually all sensory information (expect olfactory) is relayed through thalamic nuclei before it reaches cortex, and vice versa, significant thalamic input comes from cortical outputs. This reciprocal loop occurs via the Thalamic Reticular Nucleus (TRN)—a thin shell of inhibitory neurons that encases the thalamus. Within DST, the thalamus—TRN—cortex assemblage is the “engine” of consciousness which produces the oscillatory activity at baseline that comprises default space. These loops create membrane-potential oscillations (especially at alpha and gamma frequencies) that are thought to bind disparate neuronal populations via rapid, unrelenting oscillations. The TRN is key to such binding: it rhythmically inhibits thalamic relay cells to synchronize (and thus impact cortical rhythms) which determines when sensory information impinges upon cortex. The TRN has also been shown to provide burst vs. tonic-firing potentials to thalamic cells based on resting membrane voltages, allowing it to establish if information continues to flow. Therefore, it can contribute to consciousness being gated (i.e., sleeping vs. waking) as thalamus will relay sensory information or remain silent. Relative to DST's approach of “smart integration,” this means that inclusivity the TRN and thalamus ensure new, relevant sensory inputs are integrated at the right time with ongoing internal oscillations, that they will be blotted out at the wrong time, and simultaneously impose an involuntary quality of information that only makes sense under default space. (B) Descending Efferent Pathways to Sensory Organs: What makes DST unique is the inclusion of descending corticofugal (top down) pathways that project from brain back to peripheral sense organs. In many frameworks supporting a sensory system, processing occurs in a feed forward fashion from receptors to cortex (and beyond). And yet, well known efferent systems exist; for instance, 1. medial olivocochlear (MOC) fibers project from the brainstem to the cochlea to modulate hair cell responsiveness, and retinopetal fibers project from several cortical sources to the retina to modulate receptive nature of retinal processing (via various neuromodulators like histamine or serotonin). DST proposes that these descending projections are not merely adjusting information but active feedback loops engaged with peripherally existing/centrally derived oscillations. In theory, one could propose that sense receptors engaged in oscillatory activity could be entrained by cortical or brainstem instructions—essentially “tuned” by central rhythms. For example, olivocochlear output can yield coherent oscillations at appropriate frequencies (perhaps gamma or beta waves) in cochlear hair cells aligning auditory transduction with cortical prediction. Similarly, retinopetal events can define retinal adjustment via amacrine/ganglion cells in a phase dependent way relative to visual cortex oscillations engaged in attentional tasks. Note that these are speculative ideas: we know connections exist but whether they project oscillatory impulses that phase lock peripheral activity with cortical requires further empirical validation. Should this be confirmed, it would align with DST posit of a sensorium integrated bi-directionally over time (not just one direction transmission as stimuli enter through one sensor and become merely processed in cortex). Below in “Sensory Organ Oscillations and Functional Coherence” we discuss details and likelihood of such phase locking. (C) Sensory Organs as “Smart Screens”—Preprocessing and Integration Sites: Relative to passive receptors, DST positions that sensory organs are intelligent preprocessing sites—“smart screens” that project organized information from corporeal reality into the brain and intelligently integrate feedback signals. The screen aspect relates to stimuli like the retina projects a picture (or image) via organized mapping of rods and cones or frequency maps within a cochlea. They're smart because they utilize features (generally lateral inhibitory circuits or resonance characteristics) to enhance contrast, filter noise, or even retain short-term memory of acts. For example, the retina uses lateral inhibition via horizontal and amacrine cells which sharpens spatial contrast and edge detection—in a sense, computing a response long before any visual information gets to the brain. The cochlea's outer hair cells provides frequency-specific amplification or suppression—as if it's an equalizer set to sensitivity that can dynamically adjust. In DST, these operations are not reflexes in isolation but critical functionally integrated components of central processing. Thus, articulation exists as integration sites where peripheral processed information meets central processing feedback. For vision, one major integration site is the lateral geniculate nucleus (LGN) of the thalamus: it receives extensive retinal projections at its input nucleus but is massively innervated by cortex which enables contextualization from cortex (attention, expectancy) for determining progression onward. For audition, the cochlear nucleus integrates descending information from cortical processing with ascending auditory processing before cortex. DST argues that here peripheral oscillatory activity can entrain or sync with cortical rhythms for functional coherence. By exploring anatomical connections deeper, we could hypothesize that by the time information gets to primary sensory cortex, it's already been temporally aligned to the rhythm of its ongoing complexity—and this is situated through kind smart screen processing and thalamic gating. Such would enable how such impacts present on sensory cortex nuggets ideally; theories of communication through coherence suggest translation only happens when sender and receiver oscillations are phase locked. For how subjective experience becomes smooth and continuous, this explains how stimuli come and go yet blend into ongoing internal oscillatory nature without interrupted continuity of perceptual field.

(C) Sensory Organs as “Smart Screens” - Preprocessors & Integrators: Rather than being passive receivers, DST acknowledges sensory organs as active preprocessors—as “smart screens” projecting the information they process to the brain and integrating feedback signaling. The word screen matters because the retina, cochlea, skin, etc. all lay a map of information out for them to see (like a screen image or a frequency map extending along the cochlea); they're smart because they have intra-mechanisms (often lateral inhibitory circuits or resonant properties) that enhance contrast, minimize white noise, or maybe even create short-term memories of stimuli. For example, the retina is involved in lateral inhibition due to horizontal and amacrine cells that enhance spatial contrast and edge detection—a computation applied before the visual information ever reaches the brain. The cochlea has frequency specific amplifications and attenuations via outer hair cells, comparable to a type of equalizer that can be actively adjusted. In DST, such processing occurs not as reflexive functions separate from other activities but as functionally integrated with central functioning. Yet the notion of a “smart screen” implies that certain points of integration exist where peripheral activity is screened through feedback from central activation. For vision, one such point is within the lateral geniculate nucleus (LGN) of the thalamus: it receives transmission from the retina but is also massively innervated by cortex suggesting that contextualization in cortex (attention and expectation) can bias what is sent downstream. For audition, the integrative nucleus exists somewhere between the cochlear nucleus and superior olivary complex which integrates descending information from cortex with ascending information from auditory periphery. DST argues that it is here that such oscillatory activity from the periphery can entrain—with—sync with—the frequency patterns generated by cortical rhythms. Therefore, we posit that by the time sensory information reaches primary sensory cortex, it has been integrated through temporal alignment of afferent information to sustained oscillations in cortex (via “smart screen” preprocessing and thalamic integration). Such a phenomenon would facilitate the transaction of such information with cortical neurons, akin to “communicating through coherence” (where an oscillation of a producing source must be phase aligned to its communicating target for effective transmission). This is significant for how subjective experience remains sustained and integrated: as occurrences come and go, they make sense within the context of an already established active oscillatory stream without interrupting what's going on in the perceivable field.

(D) A Unified 3D Conscious Field and Testable Predictions. Therefore, all the aforementioned leads to a unified conscious experience—a 3D field (the default space) where the external world meets the internal body. Empirically, this field has spatial and temporal integrity. For example, spatial features (size, distance, location) of object experience map to oscillatory patterns in the brain while temporal features (onset, duration) map to sequential entrainment; the thalamocortical system, with the help of the body, fills in this field even in the absence of any input (e.g., the “dark” field of vision while eyefolding/binding is maintained by its resting oscillation); consciousness occurs when sense input modulates such a resting level of activity in certain ways that it fills the field with specific objects (colors, sounds, feelings) placed and dated therein. DST makes clear empirical predictions. Prediction 1: If consciousness depends on phase alignment between peripheral signals and cortical oscillations, then disrupting this alignment should impair consciousness. For instance, pharmacological silencing of olivocochlear efferents or retinopetal fibers that desynchronizes down-streaming/hierarchy connections should reduce vividness or coherence of the resulting experience. Prediction 2: If consciousness is aided by coherence, then enhancing coherence will enhance phenomena of consciousness. For instance, one can test whether vagus nerve stimulation—which entrains certain brain oscillations due to [visceral] input—affects any phenomenon of clarity, integration or binding of multimodal aspects; if controlled breathing exercises show some rhythmic pattern (as we'll see later), they can be tested for measurable impacts upon coherent clarity as well. Prediction 3: If even at rest rhythms of organs contribute to resting-state networks of the brain, we should see oscillatory correlations between central networks and peripheral systems. As we'll see in subsequent chapters, we do see phase-locked oscillative activity between central networks and cardiac rhythms and gastric rhythms—which serves preliminary support for such a prediction. Prediction 4: Metaphorically, if consciousness is an electrical trans-corporeal processing entity, then phenomena that cut the connection (e.g., high spinal cord injury) will disrupt consciousness in core content-relevant ways—but spare core awareness; we explore the HCCI prediction as a case study—DST predicts that while paralysis/indifference eliminate input (and therefore contents such as limb position) from consciousness, awareness continues, sense of self continues, and some basic functioning of some level of perceptual processing continues—accessible via thalamic relay connection with awareness and head/body input (heart and lung input). This is contrary to DST prediction for prioritization stroke, thalamic disconnection, and cortical suppression which DST predicts will greatly disrupt or eliminate consciousness altogether.

In the following sections, we delve into key aspects of DST that required clarification or correction, and we discuss relevant evidence for each. We begin with the contentious idea of long-range gamma synchrony between sensory receptors and cortex, reframing it as a hypothesis and reviewing data on sensory-cortical coupling. Next, we address the motor system example, explaining surround inhibition and how focused motor control fits into DST's framework of global synchrony. Then we explore the role of internal organ rhythms in consciousness, including what happens when those inputs are lost (as in HCCI). Finally, we compare DST's explanatory power and scope to that of other major consciousness theories, to highlight what new insights DST provides and where empirical work is needed to substantiate it.

## Synchronized gamma oscillations from sensory organs to cortex: hypothesis and evidence

Among the most bold assertions of DST is that fast oscillatory activity (specifically in the gamma frequency band, ~30–100 Hz) could fall entrained across the entire sensory neuraxis—from peripheral receptors (in eyes, ears, skin, etc.) to even the most distal forms of cortical representation. In prior iterations of this hypothesis, we stated that such an entrained coherency could exist for the sake of integration—for example, oscillations in retinal cells could be “phase-locked” to the cortical gamma oscillations so that visual experience is one long, integrated experience. Such claims were speculative, and as our reviewers suggested, potentially overreaching, as little exists to support it directly. We want to clarify, therefore, that cross-scale gamma synchrony is a hypothesis of DST but not a definitive finding that must be acknowledged as such. We put it forth as a conceptual link between what we know already (that communication through coherence exists; that temporally based coding systems exist across sensory modalities) and review concepts where this notion applies but also where findings suggest such a thing.

Clearer Hypothesis: We hypothesize that there are moments—most likely when perceiving and attending actively—when the oscillating activity of neurons in the peripheral defenders can become phase-locked, even if temporarily, to the oscillations of homologous cortical fields, thereby decreasing the effective distance between peripheral nodes and central channels into what DST would deem “functional proximity,” despite still being miles apart. In other words, when two areas can oscillate in time with one another, they can communicate as if they were part of one unit. For a peripheral neuron to influence a cortical neuron, however, their activities must be aligned on one phase; if the cortex intends on receiving an input at certain oscillatory phase, an input that arrives on phase is more likely processed (and may even be enhanced through phase-dependent gain modulation). Thus, DST submits that an act of descending control (from either the cortex or thalamus via cortico- or lemniscal-specific efferents) would be to constrain peripheral activity to fall in line with cortical oscillating processes. This does NOT mean that there is a constant 1:1 phase-locking at all times—but instead, that at times, there are transient phase-lockings that consolidate the periphery and cortex under one rhythm long enough to convey or sample information. At all other times (save for this transient phase), peripheral generators may oscillate at their own rhythms, or even different frequencies.

Supporting Notions: The notion finds some support in existing assertions. For example, Fries (2015) “communication through coherence” suggests that groups of neurons work best communicating when their rhythms (phase and frequency) are coherent with each other; while this is typically reserved for brain-to-brain communication, it seems to follow—albeit somewhat farfetched—that this could extended to periphery communication. Another suggests the existence of oscillatory multiplexing; in the lateral geniculate (for visual processing) or dorsal column nuclei (for somatosensory processing), certain neurons can encode information across multiple frequency channels at once (i.e., slower frequencies for global context of stimulus, faster frequencies for high detail); it is not beyond reason to consider that one channel could be an endogenous oscillation from cortex that marks peripheral response timing.

Empirical support and plausibility: While no one can easily measure what the cortex is oscillating at through its sense organs (the retina and cochlea have been measured more in non-human applications), there is evidence that at the peripheral or first neuron level, sense organs do oscillate/facilitate oscillatory firing and can become oscillatory in lockstep with more downstream components: (1) In vision, retinal ganglion cells exhibit burst firing in certain species at potential gamma-range frequencies. One study showed that retinal inputs can drive gamma-frequency modulation in lateral geniculate nucleus (LGN) relay cells, effectively multiplexing responses within the thalamus. According to Koepsell and Sommer (2009), thalamic relay neurons of the cat visual system carry two independent channels of information: one in the low-frequency band (comprising frequency allocation below 10 Hz) conveying information of stimulus change and another in the high-gamma (~40–80 Hz) band representing the retinal oscillation. More importantly, they showed that the retinal input was phase locked across a large swath of the gamma range in the thalamus, meaning that the timing of spikes in thalamus was related to the phase of oscillatory activity of the retina. When phases of retinal oscillation became aligned across trials, information transfer in the thalamus was greatly enhanced, suggesting that a coherent phase relationship between retina and thalamus may facilitate processing. While this study never recorded from cortex, it suggests that ocularly-driven gamma oscillations may propagate through the thalamus and ultimately entrain cortical neurons. Another recent study (Stitt et al., 2023, in mouse) shows that a narrowband gamma oscillation (50–70 Hz) propagates through the awake visual system as well, phase synchronizing local field potential activity from retina to visual cortex. Although it is too early to tell with many results yet to emerge from Stitt's work, these findings suggest that some degree of gamma synchrony within natural responses across the visual stream is plausible. (2) In audition, less direct observations have been made from periphery to cortex concerning synchronized oscillations; however, indirectly, this seems plausible. For example, when paying attention to auditory stimuli, low-frequency cortical oscillations (theta/alpha) can entrain to the rhythm of sounds to which one attends (known as phase entrainment). Through corticofugal loops, this might theoretically entrain the cochlea via the efferent system, although direct evidence has yet to emerge. What has emerged is that the cochlea hair cells can generate their electrical oscillation (i.e., otoacoustic emissions reflect this), while these hairs' gain is modulated by efferent feedback. Thus, it's plausible that in states of expectation or attention, a descending input could have biased hair cell activity at certain phases during an ongoing oscillation (associated with a beat or a self-generated rhythm), effectively entraining when the cochlea is active. Auditory brainstem response data suggest enhanced phase-coherence under high-attentional engagement as well, indirectly validating top-down timing entrainment (i.e., ear-canonical systems preferential signaling trumps operation time hints based on what cortex is determined). (1) In somatosensation, peripheral nerves can entrain with vibrations or flutter/tactile flutter, and cortical neurons oscillate at correlated beta or gamma ranges as one engages such an experience. Thus, if one experiences actively engaging one's world (whisking or exploratory touches), motor-generated rhythmic input could entrain with oscillations occurring at the cortical level that sample touch. Thus, proprioceptive feedback during such engagement could phase-lock with motor cortex oscillations (akin to how active sensing solutions emerge for predictive coding; here, however, it's about alignment along a rhythm). Overall, while we cannot conclude defiantly “the cortex and all its sensory receptors oscillate in unison,” we have pockets of evidence showing oscillatory coupling along specific dimensions: ideally retina-LGN and perhaps thalamus-cortex and likely cortex-back to thalamus/receptors through feedback loops. But DST claims these pockets are part of an extended archipelago—a globally synchronized network at the rhythm level—at least while consciously engaged. However, we must caution that distance and propagation limits simultaneously limit synchrony. A gamma-like oscillation period is ~25 ms; a signal going between cortex to receptor (or receptor to cortex) may take on the order of 10–20 ms to propagate, especially if multiple synapses are involved. For perfect phase-locking along these longer paths would require compensating for delays (it could be that there is alignment coinciding with latencies—as in, a descending signal could expect its source will take time so by the time it gets to periphery, it matches with the reality at the end phase). For such compensation measures might be easier for lower frequencies (by avoiding quicker cycles). Notably, we have great evidence for brain-body coupling for lower frequencies (e.g., respiratory rhythms, cardiac rhythms—these are discussed later). Therefore, a more empirical expectation is that global synchrony might occur hierarchically-in-nested fashion. Slower oscillations might synchronize larger brain-body operations while faster one synchronize smaller operations that can still crystallize into behaviors according to slower phases. This resonates with cross-frequency coupling (e.g., gamma rides on the phase of theta).

In summary, we have rephrased our original strong claims about cortex-sensory gamma synchrony into a testable hypothesis, supported by some empirical precedents but awaiting direct validation. We encourage future experiments that simultaneously record peripheral and cortical signals (for instance, retinal field potentials and EEG, or ECoG and cochlear microphonics) to search for transient phase-locking or coherence. If found, it would bolster DST's central tenet of a *bioelectric unity* underlying conscious experience. If not, DST may need revision to constrain the synchrony to central nervous system loops and slower systemic rhythms, with peripheral input playing a less dynamically coupled role.

## Surround inhibition in motor cortex: focusing output and preventing overflow

In past experiments surrounding DST, we compared sensory systems' methods of silencing and dominance of motor activity to confirm the need for attentional focus and cohesion across sensory and active efforts. For example, we referenced the cortical silent period (CSP)—a phenomenon resulting from transcranial magnetic stimulation—in our silencing efforts, theorizing it could align with conscious motor activity. However, this was a poor choice—CSP is a post-movement suppression that occurs after a motor evoked potential due to polysynaptic spinal and cortical inhibition and has nothing to do with spatially controlled movement. Thus, the reviewers were correct to indicate that a better comparison would be surround inhibition (SI) within the motor system as it promotes motor command by silencing surrounding populations that might otherwise activate non-targeted musculature. Therefore, we amend the literature and join the discourse by explaining SI, its relevance to the DST paradigm, particularly relative to the issue of “motor overflow” (unintentional movements with purposeful movement) and what DST might posit about these occurrences.

Surround Inhibition in the Motor Cortex: In an analogous comparison to lateral inhibition in sensory systems (which promotes sharper sensory discrimination by quieting adjacent inputs), surround inhibition in the motor system creates focus of neurological activation to represent the predetermined movement. For example, if one intends to create a fine movement (for example, flexing one's ring finger), the motor cortex activates its cortical representation of that muscle. Still, this action simultaneously happens through inhibitory interneurons (predominantly GABAergic) reducing the potentiality of adjacent representations (adjacent fingers, wrist musculature). This center-surround approach increases contrast of what's on and what's off. Thus, one flexes their ring finger with exclusion of other muscle activation effort.

Surround inhibition can be observed in humans physiologically with paired-pulse TMS or multi-electrode recordings (Beck and Hallett, 2011): when one representation is stimulated for one muscle, there exists diminished excitability for adjacent muscle representations. It's shown that skilled precision tasking invites increased surround inhibitions—for example, SI is likely greater for one's dominant hand or trained musicians, presumably to elicit fine motor control. Interestingly, those with neurological conditions that disrupt fine motor control, such as focal hand dystonia (i.e., writer's cramp), reflect decreased surround inhibition (Beck et al., 2009). For these individuals attempt to activate one muscle; they involuntarily co-activate adjacent musculature. Thus, when someone with writer's cramp attempts to flex their ring finger, they cannot help but flex their other fingers—and this is a type of motor overflow attributed to poor inhibition. Likewise, surround inhibition is decreased in individuals with Parkinson's disease who may find themselves with overflow movements or mirror movements (opposite limbs involuntarily engaged) when they try to do something—or when they sit rigid at rest, attempting to keep still. Thus, surround inhibition is necessary for functioning because without it, the motor system cannot constrain “output” from a motor plan to specific musculature, but rather generalizes the ability to move.

Motor overflow and conscious will: “Motor overflow” refers to these extra, automatic movements that occur in addition to a movement that one consciously generates. For healthy adults, overflow occurs a la bit (e.g., a child concentrating so hard on writing that he involuntarily moves his opposite hand in the same direction; someone performing a challenging action might involuntarily twitch certain digits that are not being used in that action). Over the course of one's life, the brain suppresses such overflow as an individual learns to better control fine motor actions. Thus, from a DST perspective, how does this pertain to consciousness? We could say that surround inhibition is yet another way that the non-localized nature of bodily movements in conscious awareness it constrained, such that a person always has the feeling of having only one movement as opposed to being consciously aware of various muscle contractions. For example, if someone did not possess surround inhibition, every time they attempted a fine contraction, they would have proprioceptive feedback that complicates their sense of agency—various muscles moving against one's intention, and potentially contralaterally, could frustrate one's intention and subsequent sense of agency. In other words, SI allows for a one-to-one correspondence between what one consciously wants to do (“move finger A”) and what is actually being accomplished, as it prevents feedback of another, unintended action from emerging (prohibiting “finger B” from moving inadvertently and producing unnecessary proprioceptive feedback). Within DST, surround inhibition is understood as part of the global synchrony process that complements one's concentrated exertion. When enough “certain” commands are generated, for example, a specific oscillation could occur from the relevant neurons engaged in carrying out that motor command. If the motor command is to be executed and entered into conscious awareness, it would be advantageous for the rest of the motor network (and somatosensory feedback network) to be either out-of-phase or quiescent so that they do not create overlap. Therefore, SI would render a phenomenon whereby the recruited neurons are either excluded from the synchronous, firing pattern (remaining silent) or even suppressed regarding involvement in oscillator engagement. This reflects how visual attention kept in one place suppresses neuronal responses to distracting occurrences in peripheral spaces—it's a spotlight on a focus, which DST would claim is due to selective synchrony. For motor control, it's a focus on one muscle group and SI creates darkness around it. Why it's meaningful to DST relative to conscious motor control: by including surround inhibition as part of the picture, DST has an explanation for how otherwise unified conscious space can have localized, precise content without bleeding into one another. The meaning that the motor system adds to conscious awareness is not merely that one can move, but that one acknowledges and operates upon a difference between intended and unintended movements. The very quality of awareness and agency relies on distinguishing what has happened vs. what was intended via various forms of motor commands. Surround inhibition makes sure that what has happened (at least in terms of what muscles are moving) ends up more clearly aligned with what was intended. If DST states that there is an internal representation of the body (the “body schema” in default space), surround inhibition keeps that representation's integrity while it is acted upon: only the portion of the body schema that corresponds to what the effector wants to act upon gets updated as moving while the rest of the body schema remains static. Such a phenomenon could be very important in allowing something to be experienced that has been moved while the rest of the body feels still; such notions are omnipresent in our sensorimotor reality but need neural facilitation to sustain them. Where surround inhibition is lessened in clinical disorders like dystonia or Parkinson's disease, people report their appropriate conscious experiences about their actions are diminished—e.g., thinking that actions happen “unconsciously” or that other pieces are moving “against their will.” The DST perspective would be that the default representation is thrown out of whack through asynchronization and unintentional activation. The conscious realm becomes noisier or chaotic instead, as incidental parts enter one's conscious workspace without requested command; this could undermine the quality of agency awareness. Ironically, yet unsurprisingly, the treatments known to improve symptoms for those with dystonia (GABAergic drugs/deep brain stimulation) improve such focused control because they restore better inhibition, analogous to DST's view of restored focus and improved systems.

## Sensory organ oscillations and functional coherence: clarifying “phase-locked” peripheral neurons

The phrase “neurons in sensory organs phase-locked with cortical oscillations” is a consolidation of one of the major variables of DST-driven outcomes that could be misread. We suggest a functional alignment, not that every neuron in periphery is genuinely firing in conjunction with the cortical EEG rhythms at any given point, but that there is a definable phase relationship which can be used for effective conveyance. Here, we address this assumption and keep it within anatomical confines. We explain how such valving could happen without attempting to claim some undisclosed, universal connection and why it is useful for conscious awareness.

What Phase-Locking Means in DST: Phase-locking means that the timing of spikes or oscillatory cycles in a sensory receptor is systematically related to the phase of an oscillation in a related cortical area. For example, let's assume primary visual cortex (V1) is undergoing a gamma oscillation. A firing of a retinal ganglion cell can be phase-locked if it tends to fire during a given phase of 1′s gamma cycle (and potentially via the LGN as a relay in between). Importantly, this does not require a direct physical connection from V1 to that retinal cell; it can happen indirectly through feedback constraints that dictate when the neurons in the LGN (receiving information from the retina) discharge, effectively aligning retinal driven input to phase of V1 rhythm. Thus, phase-locking can occur through a series of causative actions: cortical feedback → thalamic indirection → timing of the thalamic relay discharge → aligned retinal output during that time frame. The important part is, when examined, the peripheral input is carried to the cortex relative to a consistent phase of the cortical oscillation.

Thus, it's like an analogy to a conductor and an orchestra: the conductor (cortex/TRN) sets a beat, and various sections of the orchestra (subsets of sensory organizations) play along to that beat. Each instrument (let's say a retinal neuron) doesn't play all the time; it plays notes (spikes) at specific counts relative to that beat. Phase-locking is akin to making sure the instrument plays at the precise time it should come in. The anatomical pathways (feedback loops) carry the conductor's beat down to the orchestra. If each section abides by the conductor's direction, their outputs are aligned with each other as well and thus produce harmonious sound (integrated perception).

Limits of Anatomical and Functional Locking: There are limits to this locking. Peripherally derived neurons are heterogeneous and quite often operate independently from one another (e.g., photoreceptor oscillations occur mostly due to light entrainment). The long-distance phase-lock likely occurs transitorily vs. continuously. Physiologically, it may only occur during certain brain states (e.g., focused attention, anticipation) when top-down signals can dominate features and noise is resolved. In addition, phase-locking may only involve certain frequencies with respect to oscillation. Fast oscillations (gamma) exist locally and may not exist across long distances without intermediate relays; slow rhythms (theta, alpha), however, may better unify distant regions since one oscillation may last longer in time (50–100 ms) allowing for signal transduction along with realignment. Indeed, evidence suggests general coherence occurs in slow bands throughout the brain but sometimes extends to peripheral rhythms like heart rate or respiration; thus it is anatomically feasible that cortex could phase-align peripheral components at least at those slower paces. For example, alpha-band (8–12 Hz) oscillations are known to influence sensory excitability; thus a cortical alpha wave could influence excitability of thalamic relay neurons periodically which also gates when information from the retina can reach it. Retinal cells can then properly convey at bursts when at alpha phase openings of gates.

Furthermore, delays and latencies mean that “in phase” does not necessarily equate with “at the same time.” A consistent phase relationship can occur where the peripheral spike lags behind the cortical oscillation by a fixed number of milliseconds (a phase offset). Consistency is key, not absence of lag. This is what we mean by coherence—not coherence of true synchrony (zero phase difference). Coherence exists throughout many interactions within neuro-neuro interactions (e.g., frontal and parietal cortex in theta band), we extend this idea to brain-body interactions.

Functional Coherence: Why would phase locking be important for awareness? If sensory information is entering out of random phases relative to cortical oscillations, a lot of the time, it'll enter at less excitable phases (neurons are refractory or inhibited), and thus, it won't be able to be optimally perceived/synthesized. By biasing input to an excitatory phase, the brain ensures that the signal is there in the most meaningful way it can be, thus making that sensory experience more salient or clearer in awareness. Coherence also facilitates different sensory modalities coming in at the same time and being able to make sense of each other. For example, vision and audition need to be integrated (lips moving vs. sounds). If visual and auditory elements are phase locked to the same frequency (the low-frequency beat associated with attentional or mouth movements), then their integration at the multimodal cortex is easier, avoiding decoupled onsets and enhancing percepts of simultaneity (the ventriloquist effect or binding of speech). Another notion of functional usefulness is predictive coding: if the brain sends predictions downward, those predictions need to arrive with a rhythm/timing. If the sensory apparatus aligns with those predictions, any deviance (prediction error) emerges as a phase desynchronization or unmodeled blip, which can update the model. Within DST, the downward oscillations would relate to predictions and phase aligning would ensure that the real incoming arrivals align with them unless something novel happens (which causes the desynchronization, suggesting surprise). A Peripheral-Central Example- Olfaction and Breath. One clear case of peripheral-central phase locking coherence is olfaction. Olfaction is unique as connected to a peripheral rhythm; unlike other senses, olfactory stimulation is intimately connected to one's breath. In rodents, sniffing (~4–10 Hz) establishes olfactory bulb and piriform cortex oscillations. In humans, nasal breathing temporally entrains limbic oscillations—power in amygdala and hippocampus occurs with inhalation and exhalation phases. Here the peripheral oscillation (nasal breathing) effectively phase locks the activity of breathing to the oscillatory activities of multiple neural regions. As such, cognitive functions such as discrimination of memory/emotion fluctuate relative to breath phase (better during inhalation in one study). This is an example of a system-determined oscillation that imposes a temporal structure to which the brain “goes along with.” It's an extreme example of coherence between mind and body since it's so easily assessed; breathing is global and relatively slow. DST would predict that even more microscopic or faster signals (in retina or cochlea) could likewise impose temporal constraints to which the brain aligns itself for different functional purposes (for things like intensity of stimulation vs. aligning with breathing as a metabolic frequency). Neurons within by referring to it as a “network of sensory organs phase locked with cortical oscillations,” we acknowledge the integrative role via periodicity. This is not to say a photoreceptor turns voltage at 40 Hz, and a V1 neuron turns at 40 Hz for 3 h a day, constantly communicating. Instead, there are times—when stimulation is provided, or attention is needed—that the photoreceptor output (dynamically changed due to the challenge of retinal and thalamic processing) will be accessible during the preferred phase of the V1 local field potential oscillation generator in relation to the phasic timecourse across longer timescales. In other words, it's like “tuning in” to a station. The frequency of the carrier wave aligns, and the signal can come through. We acknowledge that this is somewhat speculative beyond what is known from peripherally established anatomy, but it does not overreach. It acknowledges known feedback loops, oscillatory dynamics—and one could validate such a hypothesis by determining whether fine-temporal dependences exist between peripherally initiated signals—EEG-detectable retinal potentials or OAE-detectable cochlear potentials—and central, EEG-detectable rhythms across tasks and states of consciousness. The emerging phenomenon of multi-site recording and subsequent phase coupling may determine whether such a discrete alignment exists. If validated, it would imply that DST was correct in asserting that a closely integrated brain-body oscillatory network exists, facilitating operations toward conscious awareness. If disproven, DST will need to be revised so that awareness-based synchrony exists only within central circuits and slower, homeostatic-type rhythms.

## Interoceptive rhythms and consciousness: contributions of heartbeat, respiration, and gastric waves

Beyond the canonical five senses, an additional aspect of DST is interoception—the understanding and awareness of internal states of the body—as vital for conscious awareness (Critchley et al., 2004). For example, the body's viscera generate rhythms: the heartbeat (~1 Hz in humans), breathing (~0.2–0.3 Hz in resting adults), gastric slow waves (~0.05 Hz) represent some of the most powerful. They function as unending inputs to the nervous system—via neural (e.g., vagus nerve, spinal visceral afferents) and humoral pathways. Not only do we extend our considerations of how such signals affect our consciousness, but we acknowledge that more and more research suggests brain networks are entrained—and even coupled—to such bodily rhythms. Finally, we discuss how DST explains why patients can be conscious despite high cervical cord injury and extensive other conditions where such signals are lost; from the perspective of DST, it still exists as a default state of consciousness—with only its accompanying contents or schemas lost.

Heartbeat and Cardiac-Brain Coupling: The cardiac cycle is a fundamental physiologic rhythm of life. Each heartbeat represents a rise in blood pressure which is registered at baroreceptors and relayed to the brain (predominantly brainstem via vagus and glossopharyngeal nerves) (Craig, 2009; Damasio, 2010; Park and Blanke, 2019). Thus, there exists a phasic signal time-locked to each heartbeat that may enhance or modulate cortical activity. Researchers have identified heartbeat-evoked potentials (HEPs) within the EEG—small fluctuations of electrical voltage which occur after each heartbeat, often investigated in the field of interoceptive awareness. Intriguingly, they seem to be connected to conscious experience, as well. For example, the amplitude o r latency of HEPs is related to how well participants can feel their heartbeat and is indicative of self-related processing in the midline cortical structures. From a DST perspective, the heartbeat is a continuous rhythmic oscillation (~1 Hz) that the brain not merely detects but incorporates into its oscillatory hierarchy. It may act as an “anchor” for consciousness—a rhythmic sound-like background that informs how we perceive Being and Being alive. Such revelations support that heart-brain integration is essential for consciousness. For example, Candia-Rivera (2022) finds that in patients with disorders of consciousness, thresholds of heart-brain coupling (for example, timing relations between beating hearts and brain responses) determine residual consciousness. Additionally, data show that heart-brain coupling (in terms of HEPs) predicts perceptual consciousness and sense of self for those with intact consciousness. Thus, this signal is part of the integrated conscious experience—that is, a continuously anticipated interoceptive context that can bias and modulate how we perceive external reality (e.g., stimulation received during systole—which involves excess baroreceptor firing—may be perceived as slightly weaker than stimulation received during diastole due to transient cortical suppression via baroreflex feedback). DST integrates this by asserting that such oscillation exists as part of the baseline of default space. The rhythmic “pulse” makes up the infrastructure of temporality experienced within a felt inner world—it contributes to a sense of time feeling continuous. It also renders rhythmic input to which other oscillations may align (there's evidence for cross-frequency coupling between heartbeat and delta/theta EEG oscillations). Ultimately, from a bodily perspective, one's heartbeat reminds one of existence. We lose consciousness practically instantaneously during cardiac arrest—no more blood flow—but also no more of that informational drive.

Respiration and Rhythmic Breathing: Breathing is both autonomic and voluntary, meaning this rhythmic phenomenon is quite unique as it occupies the space between subconscious and conscious. It's no wonder that many meditative programs and mindful practices focus on breathing, even if implicitly, suggesting an awareness of breathing's relationship to mind states. Neuro-physiologically, breathing is extensive: it entrains oscillations in olfactory circuits and limbic circuits discussed above and within the neocortex, the respiratory rhythm is found to couple with our own brain oscillations; for example, Karavaev et al. (2018) found respiration-entrained brain oscillations detectable via fMRI BOLD signals. Moreover, Zelano et al. (2016) find that nasal breathing (not mouth breathing) entrains oscillatory power which relates to cognitive performance in humans. In another study, researchers concluded their findings based upon rhythmic breathing's hierarchical organization of neuronal oscillations across the entire brain—like a composite pacemaker for mind and matter. From DST's perspective, breathing exists as a temporal layer of oscillatory organization to default space: it breathes slower than alpha waves and faster than circadian rhythms; Dotov notes it correlates to a middle layer of organization (the “respiratory oscillatory mode” may relate to emotional matters and memory). In previous endeavors, we found a connection between respiration and something we called a “mental atmosphere,” meaning people's moods and abilities to think clearly fluctuate as they breathe (note how deep breaths induce calmness while their attenuation speeds provoke anxiety). Some of this can be accounted for by physiology (CO_2_ levels, fight or flight response), but DST deduces a further mechanism: the respiratory-brain synchronicity contributes to regulating brain dynamics. This means that synchronicity within limbic circuits helps with vigilant integration of emotion into awareness or memory gating. Besides, being aware of one's breath (as with meditation) may increase the brain's relationship to this body-induced rhythm which accommodates a more unified state of awareness with brain and body (often referred to as “centered”). Empirically, this is supported by studies which suggest certain memory tasks are influenced by breath timing—some people remember better at particular times during breathing. DST can explain these occurrences by acknowledging the respiratory rhythm encourages certain theta-band oscillations in the brain (the nasal rhythm registers at ~0.16–0.33 Hz. Yet intriguingly, it encourages faster oscillations in olfactory cortex at 2–12 Hz due to air-flow dynamics which then radiates through the rest of the system). Thus, breathing is doubly neurophysiological: as a slow modulator, and a fast entrainer via smell. Both factors contribute to the experience of consciousness (olfaction is related to emotion, likely because of this). Gastric Slow Waves and the “Gut-Brain” Rhythm: one of the most extraordinary new findings relevant to DST is that the stomach has an electrical rhythm (approximately 0.05 Hz; one cycle every 20 s) detectable within brain functioning as well. For example, Rebollo et al. (2018) found a resting-state network phase-synchronized across human brains to the gastric basal rhythm; combined electrogastrogram (EGG) with fMRI suggested that activity within somatosensory cortex, insula, and visual cortex waxed and waned with the stomach's slow wave identified as the “gastric network.” In other words, the brain does not passively receive signals from the stomach, but instead, actively represents its alignment to its own body's gestational rhythm. While still unclear what this would mean functionally, many of these regions are involved in forming interoceptive bodily maps and exteroceptive maps meaning the energy from the gut could impart knowledge about bodily self-representation or peripersonal space. One potential benefit from the slow 0.05 Hz oscillation is it acts like a carrier frequency for integrating long-term bodily information or possibly crowds out the slow fluctuations from default mode network. For DST, this vigorous correspondence serves as strong evidence for embodied consciousness: a visceral organ oscillation is measurably part of the brain's activity. In a way, one might say the gut is our second brain (enteric nervous system); while no conscious thought arises from the gut, it could very well influence the conscious state (anyone who's ever become “hangry” knows how disposition can change; perhaps the gastric rhythm is one subtle contributor). Another example cites a connection between the phases of the gastric cycle aligning with hippocampal theta and then behavior on memory tasks; if the stomach and brain are aligned at some point, maybe cognition benefits from it. DST can incorporate this by asserting that the gastric slow wave acts as a baseline tempo for what goes on “in here,” in brain activity through vagal afferents from the stomach to brainstem and onward to cortex (the connection is non-controversial). In our multilayer cafe of consciousness, we could associate gastric oscillation with baseline unconscious/autonomic processes along with slow cortical oscillations and default mode network activity to set the stage for which faster occurrences build content of consciousness.

## High cervical cord injury (HCCI) – preserved core, altered content

Now, we have a specific condition to test DST's integration of interoceptive rhythms: high cervical spinal cord injury. With HCCI (for example, complete transection at C1–C2), the brain is effectively severed from the body a neck down. The individual loses all somatosensory (touch/proprioception/pain) and motor input and output below that level—and the ability to autonomically regulate those areas is impaired. Yet this person is fully aware (absent head injury)—they can see, hear, think, and feel emotionally. So why does consciousness not dissipate or fundamentally shift if it's such an integral part of brain-body action and engagement when the body cuts off? Elevation of DST occurs due to differentiating between the core of consciousness vs. the content (or schema) of which it's made up. According to DST, while the core is likely preserved, the content is significantly altered. Preservation of the core means that the most fundamental form of consciousness being present is the fact that there exists a unified expanse of environmental understanding with some first-person perspective (referred to as core or primary consciousness at times). The content/schema is what exists within that realm that makes it so complicated—like representing one's body, the established sensory reality at any given time, one's thoughts, etc. For DST, HCCI preserves the core because what needs to be operationalized in the brain to punch in oscillatory rhythms remains the same; some required bodily inputs remain. The brainstem and cranial nerves remain intact—for example, the vagus nerve (cranial X) can still transmit data from thoracic and abdominal organs (heart, lungs, pieces of gut) to the nucleus of the solitary tract; vagal techniques can still communicate with blood pressure; somatosensory input can still extend from above injury structures (neck, face, head). In addition, having a functional brain with intact thalamocortical loops can support a default brain—even if it's a more vacant one. Therefore, DST would say the core is psycho-consciously preserved. People with high spinal injuries report consistent awareness, and any disruption to consciousness has little to do with spinal cord injury but other factors externalized beyond brain-body awareness. This connects to James-Lange theory expectations partly validated; early theorists said that without somatic feedback, either emotions or great determination would be decreased because of disconnection and lack of importance. Yet many high SCI show little/low capability of diminished potential for emotional feeling (although they may deal with emotional expression and sympathetic arousal). Clinicians note a variety of depression in these individuals due to psychosocial complications (they can't usually change their quality of life post spinal injuries), yet they don't fall into a vegetative state based purely on body feedback loss—that would say that the brain alone is sufficient for core consciousness—which DST would assert. What is altered in HCCI is the schema: namely, body and associated sensorimotor schema experiences. The person cannot feel their body below their neck anymore; they often acknowledge a disconnection or that something “isn't there” beyond a particular point to their awareness. They may still have a visual representation of their body—or phantom sensations—but over time, these disappear or change, as no newer afferent signals can ground them. For DST, this part of the default space that once composed info from the torso and limbs goes quiet or filled in by memory alone. The conscious self is largely “a head in a jar”—so to speak—where vision, hearing, and neck/face sensitive socio-political realities are the most significant grounded integrated spatial experiences. Interoceptive rhythms like heartbeat and breathing help—perhaps asserting how these people still have significant psychological awareness and emotional states (the heartbeat still echoes; minimal gut movement occurs via redundant vagus nerve) but stating specific conscious ideas lacks great cognitive capacity based on what's good from sensory information alone at this juncture. Therefore, this is how the scope of conscious content minimizes. This supports DST's prediction that when people connect less to their bodies, they grow in their minds excessively conversationally rich (not in intelligence or awareness but as somatic being presence necessarily decreases—and potentially emotional tonal complexity suffers). Some studies set up emotional experiments and discover subtleties; for example, they note higher reliance on cognitive appraisal than gut feels or altered activations of insular cortexes during emotionally responsive tasks. This suggests that positive sensory feedback responds differently (higher response rates)—they exist differently than expected when heart rates up against wounds translate to agitations like anger. This agrees with DST: as core valence of emotions fundamentally remains clear but how the body determines how it interacts with it is limited (i.e., increased blood pressure for fright)—the piecemeal conscious emotion lacks weight (although evidence disagrees with this being a James-Lange contribution). Ultimately, HCCI provides an instance where we can identify DST's claims. DST would claim the following:

(1) The preserved consciousness noted by HCCI supports that at least a functioning brain for itself and whatever part stressed atmosphere still connected could maintain this conscious state—we make no claim that consciousness needs a body but a lot better serves it and fills it out much better.
(2) The distinctions in conscious content (like what's given mind was via body's incapacity) illuminates action of bodily oscillatory rhythms as natural means of something like normal consent content. Where before tons of somatic rhythms operated as integrated edges (from feedback via muscle joints skin connectivity); now few—and thus while fields remain—I predict emptier ones more reliant sense and sound comes by satisfying itself present away from itself now more than ever. We predict consciousness events highly reliant on bodily feel are gone or substituted by ideas about them—but cognitive health should remain well—and prominent. Indeed many high SCI say they live “in their head” far more—this reconciles with the DST model leaving nearly all clientele default space via head when body cannot appreciate anymore.

Thus, DST can account for HCCI without contradiction: it necessitates refining our claim to “the brain-body network generates consciousness” to a recognition that the brain is the lead generator (and can do so solo if needed), but the body normally is a co-contributor that enhances and structures the experience. This nuance is important to state explicitly to avoid the misunderstanding that DST implies someone without a body (or cut off from body) would lose consciousness—they do not, though their consciousness is altered in specific ways.

## Discussion

In this revision, we have endeavored to clarify the theoretical propositions of DST (Default Space Theory) and ground them in current empirical knowledge. We addressed reviewers' concerns by tempering speculative claims, correcting factual inaccuracies, and explicitly delineating which assertions are hypotheses vs. established facts. Here we summarize the status of DST's main claims, highlight what remains **theoretical**, and outline concrete **empirical predictions** that derive from DST (some of which are already being borne out by new research). We also discuss the broader implications of DST for understanding consciousness and its disorders, acknowledging limitations and future directions.

### Theoretical vs. empirical status of key claims

Global Brain-Body Synchrony: The central claim of DST about consciousness deriving from oscillatory coupling between brain and body is partially theoretical partially empirical. The empirical part is that there are a number of studies showing biological rhythms are quasi-periodically related to brain activity (cardio-electrophysiological coupling, respiration-entrained oscillations, gastric-brain phase-locking). Thus, the notion that bodily influences engage brain rhythms in quasi-periodic/systematic fashion (as opposed to random) is supported. Yet to what extent (and importantly, the higher frequencies attributable to peripheral receptors) is not yet substantiated which makes it an active hypothesis. As for gamma synchrony being acknowledged throughout the entire cortex and periphery, this has been reconceptualized as a theoretical extension of known understanding with no current direct support.

Thalamic Hub and 3D Default Space: The supposition that the thalamus is a hub and that the TRN generates a three-dimensional default internal space is mostly theoretical; it's pieced together through knowledge of neuroanatomy and phenomenology. What is known is that the thalamus is critical for arousal and that thalamocortical loops facilitate brain rhythms (eg., sleep spindles, alpha waves) and it's theorized that this gives rise to a sense of 3D dimensionality in neuronal processing. This does not say it's implausible (eg., the brain has spatial maps, the brain can visualize 3D things so why not?) but there has not been an experiment to demonstrate that there is a “3D matrix” of oscillations in reality. Our study posits it as a model prediction—eg., DST would predict during explicit 3D mental imagery tasks or following recovery from neglect like spatial neglect, there would be specific patterns of synchronization around the thalamus and parietal regions of coordinates.

Sensory Smart Screens with Feedback Integration: The existence of lateral inhibition and local preprocessing is empirical (retinal processing and cochlear mechanisms are covered in any textbook). The extension that DST brings—that these phenomena operate as components of a conscious screen regulated by feedback—is hypothetical. There is indirect support (attention can slightly change retinal signals, olivocochlear efferents change auditory perception) but to what extent remains unknown. This is something testable: whether disrupting a smart screen mechanism changes the quality of conscious perception (does pharmacologically disrupting lateral inhibition in retina change conscious perception of contrast? very likely on a rudimentary level although subtle in behavioral impact).

Surround Inhibition and Motor Consciousness: The inclusion of surround inhibition as a component of DST is supported by decent neurophysiology and we included it to clarify DST's definition of what it means to will something motorically. This contribution is more a conceptual elaboration than a new claim about reality. It does not need to be substantiated that SI exists—it does—but what DST claims is that SI improves clarity of what is consciously willed as motor intention. While this plausibly exists, it's not strictly supported; studies can determine whether surround inhibition facilitates or minimizes overflow to determine suggestive patterns in new concepts like phenomenological reports or sense of agency.

High Cervical Injury - Core vs. Content: The fact that consciousness exists in locked-in/high SCI individuals is empirical (clinical phenomenon). The ability for DST to explain why—core vs. content distinction and the fact that preserved core suggests oscillatory circuits still operating—is interpretive. Partially substantiated by neuroimaging of such groups: DST would say their default mode network and thalamocortical activities align with non-disabled persons (core preserved) but connection patterns associated with somatosensory integration or insular interoceptive networks may be distorted (content lost). Some neuroimaging studies of SCI show reorganization in sensorimotor cortices and insula, supporting content matrix loss, while networks associated with executive functioning are in-tact—supporting retention of content. This realm needs further investigation. As a prediction, DST posits that those with HCCI will have relatively normal EEG oscillations (alpha, etc.) but perhaps differences in how coupling occurs with peripheral signals.

DST vs. Other Theories: Our comparative assessments in the previous section are theoretical but not yet determined by data (Fingelkurts et al., 2010; Jerath and Beveridge, 2018; Jerath and Crawford, 2015). For example, this inclusion does having the signals from the body as part of consciousness increase the extent of consciousness or is it epiphenomenal? The fact that brain-heart coupling and brain-gut coupling associates with levels of awareness suggests it's not epiphenomenal but we need causal experiments (like perturbing those signals predictably altering conscious states). Many fields are moving toward assessing multi-system intersections (eg., using biofeedback to alter one's heart rhythm seeing cognitive consequences) which ultimately can ascertain or disprove DST's claims about embodiment.

### Empirical predictions and ongoing tests

DST generates numerous testable predictions; here are some of the more salient aspects and their support by evidence currently:

1. Organ-Brain Coherence and Conscious Level: DST predicts that coherence will be decreased between brain rhythm states and organ dynamics in decreases in consciousness (sleep, anesthesia, comas). Some evidence: coherence in heart-brain synchrony decreases during deep anesthesia and increases partially during REM sleep (where consciousness of dreaming is evidenced). We found evidence that those in vegetative states had lower complexity of cardiorespiratory-brain dynamics than those in minimally conscious states, yet who exhibited multicentric complexity. Encouraging, but not a systematic approach. This could be tested by measuring EEG and peripheral signals at the same time and computing coherence/phase-lock measures across different levels of consciousness. 2. Increased Brain-Body Synchrony Enhances Conscious Experience: For example, regular slow breathing (which promotes baroreflex and HRV oscillation increase) should predict behavioral changes in conscious experience (increased awareness, calmness). This occurs anecdotally and is scientifically studied (mindfulness and interoception). If we could operationally define effective information integration in consciousness (through neural signal diversity or complexity, perhaps), DST predicts that it would increase as synchrony was engaged in conscious subjects. 3. Feedback to Sense Organs Improves Perception when Coherent: One pure experimental test would see whether a device could provide oscillatory stimulation to a sensory organ in lock vs. not lock with a brain rhythm and how perception would change as a result. For example, drive the retina with a flicker at a phase locked to a person's alpha oscillation to mis-locked, and see if detection threshold or qualia differ. Or use auditory clicks locked to phases of ongoing cortical oscillations (ergo, there is research that demonstrates detection is phase dependent; DST would say that is because the brain oscillation is the combined expectation pattern of brain and ear). 4. Conscious Experience in SCI with Artificial Input: Another, more futuristic experiment: if we could present SCI patient's spinal rhythm feedback from a neuroprosthesis that behaves like spinal feedback or spinal rhythm entrainment, DST predicts this would “enrich” their experience of consciousness in a bodily way. Even more simply, giving SCI patients vibrotactile stimulation that looks like a heartbeat might re-assimilate some effect of rhythm in their body—do they feel more inclusive or attuned emotionally? We have no data on this yet, but it's a theoretically explored nuance in neurorehabilitation. Implications for the Science of Consciousness: DST champions the notion that understanding consciousness requires the organismic evolution as a whole. It's not about just considering the cortex or even the brain; it blends neurologic and physiologic awareness. Therefore, clinically, those with disorders of consciousness or psychiatric disorders show us that stimulating periphery systems (such as vagal nerve stimulators for depression or breathing exercises for anxiety) may be working to change conscious dynamics vs. just calming the body. Our model explains why this body-to-brain permutation works mentally—it recalibrates oscillatory integration in the default setting. It also implies new avenues for exploration—for example, those with DOC after brain trauma may find benefit from attempts to enhance heart-brain coupling to see if it re-establishes consciousness (indeed, some are exploring cardioacceleration or changes in breath as stimuli for coma patients). Another arena concerns AI and consciousness: if one were to create a conscious agent, DST implies you'd have to give them more than just a silicon brain—maybe somebody; or at least some analog of the interoceptive rhythm. It speaks to incarnate AI arguments that disembodied intelligence will never have true phenomenal consciousness.
2. Limitations: DST is a grand theory of everything and if one isn't careful, it can become unfalsifiable. We attempted to cut out details above that would make such an untestable theory occur. It's not a formal model yet—unlike IIT, DST is descriptive, DST could learn from a mathematical formalization of its implications (i.e., network model of coupled oscillators with body nodes) to yield quantitative predictions (which frequency couplings produce optimal integration, etc.). In addition, DST does not resolve the “hard problem” of consciousness *per se* (why do oscillations feel like something)—it's merely a suggestion of the structure and dynamics of the putative neural correlates of consciousness. It results in a pragmatic identity: we are what it means to be a dynamically synchronized brain and body. Whether that's a compelling identity or not is probably dependent upon one's philosophy; what we can assess empirically are at least the correlations.

## Conclusion

Since submission, we have updated the paper to include new evidence and acknowledge concerns raised but at the root, the proclaimed notion remains that consciousness is a bioelectric phenomenon deriving from what it means to be a dynamically synchronized brain and body. The mind does not exist apart from the body; it is an orchestra with the body as conductor. Consequently, the bioelectric synchrony theory (DST) stands at the nexus of neuroscience, physiology and phenomenology; it is a compelling theory with much to be done cross-disciplinarily. We specify what elements of this theory are known (e.g., existence of brain-body coupling), what are plausible extensions of contemporary science (e.g., ramifications on function of such coupling with respect to content of consciousness) and what are more speculative (e.g., predominance of gamma synchrony to sensory periphery). The goal of this specification is to inspire particular efforts of experimentation—all of which will support, clarify or falsify DST. Whatever the results of such efforts, however, should provide clearer understanding of how the oscillating, living body leads to the conscious mind.

## Data Availability

The original contributions presented in the study are included in the article/supplementary material, further inquiries can be directed to the corresponding author.
